# Nutritional Differences between Two Orangutan Habitats: Implications for Population Density

**DOI:** 10.1371/journal.pone.0138612

**Published:** 2015-10-14

**Authors:** Erin R. Vogel, Mark E. Harrison, Astri Zulfa, Timothy D. Bransford, Shauhin E. Alavi, Simon Husson, Helen Morrogh-Bernard, Santiano, Twentinolosa Firtsman, Sri Suci Utami-Atmoko, Maria A. van Noordwijk, Wartika Rosa Farida

**Affiliations:** 1 Department of Anthropology, Rutgers University, New Brunswick, New Jersey, United States of America; 2 Department of Geography, University of Leicester, Leicester, United Kingdom; 3 The Orangutan Tropical Peatland Project, Palangka Raya, Indonesia; 4 Fakultas Biologi, Universitas Nasional Jakarta, Jakarta, Indonesia; 5 Centre for the International Cooperation in Sustainable Management of Tropical Peatlands, Palangka Raya, Indonesia; 6 Anthropological Institute and Museum, University of Zurich, Zurich, Switzerland; 7 Research Center for Biology, Indonesian Institute of Sciences (LIPI), Cibinong-Bogor, Indonesia; Texas A&M University, UNITED STATES

## Abstract

Bottom-up regulatory factors have been proposed to exert a strong influence on mammalian population density. Studies relating habitat quality to population density have typically made comparisons among distant species or communities without considering variation in food quality among localities. We compared dietary nutritional quality of two Bornean orangutan populations with differing population densities in peatland habitats, Tuanan and Sabangau, separated by 63 km. We hypothesized that because Tuanan is alluvial, the plant species included in the orangutan diet would be of higher nutritional quality compared to Sabangau, resulting in higher daily caloric intake in Tuanan. We also predicted that forest productivity would be greater in Tuanan compared to Sabangau. In support of these hypotheses, the overall quality of the diet and the quality of matched dietary items were higher in Tuanan, resulting in higher daily caloric intake compared to Sabangau. These differences in dietary nutritional quality may provide insights into why orangutan population density is almost two times greater in Tuanan compared to Sabangau, in agreement with a potentially important influence of diet quality on primate population density.

## Introduction

Bottom-up regulatory factors (e.g. nutrient availability, diet quality) are proposed to exert a strong influence on mammalian population density [1–7]. Studies relating habitat quality to primate population density have typically compared species or communities from distant sites [1,8–10], and the few studies that have quantified habitat quality at small spatial scales have examined food abundance without considering the energy available in food among the different populations [11–16]. Recently, research on two different mountain gorilla populations found that differences in habitat-wide energy availability did not lead to difference in energy intake except during the high fruit period [17]. This is not surprising given that these two populations consume food items of similar nutritional content [18]. Those studies that have examined the relationship between the nutritional composition of diets and population density in primates have focused mostly on folivorous primates [9,19–23], with very few studies focusing on frugivorous primates [24,25]. However, given that the majority of primate species are primarily frugivorous [26,27], fruit is typically much more energy rich than leaves [28–30], and different levels of reliance on high quality fruits impacts primate densities [31], studies focusing on primates with more varied diets may provide greater insights into the influence of bottom-up factors on primate populations.

Elevated nutritional requirements associated with gestation and lactation are a major limiting factor to female reproductive success in mammals [32–34]. Among primates, increased access to high quality food resources has been linked to reduced inter-birth intervals (IBI) in wild long-tailed macaques [35], baboons [36], langurs [37], and chimpanzees [38–41]. Captive orangutans have been shown to have shorter IBIs relative to their wild counterparts, presumably due to consistently high nutritional intake [42]. In general, orangutans have extremely slow life histories, with the longest inter-birth intervals, lactation periods, and juvenile dependency of any non-human primate [43–47]. Thus, all else being equal, variation in the nutritional quality of the diets of orangutan populations can result in increased reproductive success among females and ultimately impact population density [44,48].

The Bornean orangutan, *Pongo pygmaeus wurmbii*, provides an ideal species to study the relationships between dietary quality and population density. While primarily frugivorous, orangutans consume a variety of other plant items in their diets including insects, leaves, cambium, flowers, and other items [29,49–51], which vary in nutritional composition and energetic returns [28,29,52]. Bornean orangutans show dramatic fluctuations in daily caloric intake in response to fruit availability, entering into negative energy balance states during low fruit periods [29,52,53]. Furthermore, Bornean orangutans are not subject to notable mortality risk from predators [54], other than humans [55], thus diminishing the effect of natural top-down predator regulation of population density. Orangutan population density has been shown to vary markedly across and within habitat types and, while fruit productivity is thought to influence density [56,57], the proximate factors underlying this variation have not been investigated.

The tropical peat-swamp forests of Central Kalimantan, Borneo hold the largest remaining populations of orangutans [58–60], and show less pronounced fluctuations in fruit availability relative to dryland and riverine forests [3], as they do not experience the mast fruiting events that occur in more Dipterocarpaceae dominated dryland forests in the region [61]. The majority of peatlands in southern Borneo are ‘ombrogenous’, meaning that they acquire their nutrients almost exclusively through aerial precipitation and plant nutrient availability is low, as a product of this plus the typically flooded conditions [62,63]. In Sabangau, tree height, flora and fauna diversity, and orangutan density are known to vary in relation to peat depth and subsequent flooding regimes, which is thought to be linked to differences in peat nutrient availability [58,62,64]. However, there is great geographical variation in peatland ecology in Indonesia, with both peat depth [65] and the level of nutrient influx from river flooding varying substantially in the region [62].

Here, we compare the nutritional composition of plants consumed by two populations of wild orangutans in two distinct peatland habitats. The Sabangau Forest, including the research camp location, is a true ombrotrophic bog with limited nutrient input from aerial precipitation only. While the majority of the Mawas Conservation Area is similarly ombrotrophic, the Tuanan research station is located in a seasonally rheotrophic part of the area, with both shallower peat and a more alluvial flooding regime [66], and thus receives nutrients from seasonal river flooding, in addition to aerial precipitation [62,67]. In isolation, these differences would both be expected to result in higher forest fruit productivity in the Tuanan research area (cf. [62]). Because these two sites are also geographically close and climatically similar [68], any observed variation in fruit availability is likely due to differences in nutrient availability arising from these environmental differences. Finally, orangutan population density estimates between these two sites differ substantially, with Sabangau’s density estimated at 2.3 individuals/km^2^ [56] and Tuanan’s estimated at 4.3–4.5 individuals/km^2^ [56,69].

To explore if orangutan population density is related to variation in plant macronutrients and forest productivity, we systematically compared the nutritional composition of foods consumed by orangutans in these two Bornean peat-swamp forest sites. We predicted that (1) the nutritional composition of plant species in the site with shallower peat, a more alluvial flooding regime, and higher orangutan population density (Tuanan), would be of higher quality, resulting in (2) higher daily caloric intake in that site. In addition, we predicted that (3) forest productivity, measured in terms of the percentage of fruiting trees, would be greater in Tuanan.

## Materials and Methods

### Study Sites

Data were collected in the Sabangau (2° 19’ S and 113° 54’ E) and Tuanan (2°09’ S and 114°26’ E) research stations in Central Kalimantan, Indonesia, which are separated by 63 km and two major rivers (S1 Fig). The orangutan study grid in Sabangau has a peat depth of 1–4 m [62] and is located within the 500 km^2^ Natural Laboratory of Peat-swamp Forest (NLPSF), in the north-east of the 6,300 km^2^ Sabangau Forest. The Tuanan study area has a peat depth of 1–2 m in most parts, and overall ≤ 3.5 m [70], and is located in the 3,099 km^2^ Mawas Conservation Area, along the Kapuas Murung River. Both sites are recovering from selective commercial logging during the 1990s and subsequent illegal logging until the early 2000’s. Tree species composition in both sites is similar, although absolute and relative abundances of different species vary between the sites [71].

### Orangutan Behavior

Data were collected on wild orangutans (*Pongo pygmaeus wurmbii*) at both sites from 2003–2010 following standardized orangutan protocols [72,73]. Although these populations are separated by two rivers and are genetically distinguishable subpopulations, they both belong to the Central Kalimantan population of *Pongo pygmaeus wurmbii* [74,75]. Focal-animal sampling was used and only data from all-day, nest-to-nest follows on independent, adult, habituated animals were included in the analyses (Sabangau n = 636 (6977 hrs.); Tuanan, n = 2233 (25,505 hrs.); see S1 Table). Feeding rates (number of items consumed /minute; area of cambium and phloem consumed/minute for bark) were collected from August 2005-June 2007 at Sabangau [52] and July 2005–2010 at Tuanan. Feeding rates did not differ between sexes in Sabangau [52] or Tuanan (Wilcoxon paired signed rank, S = -16.5, n = 33, p = 0.79; paired by plant species and food item) and thus average feeding rates obtained for other age-sex classes were used when computing energy intake if no data were available for a particular age-sex class (see below). From the standardized data, we calculated total minutes feeding on fruit and total follow length for each focal animal. Observational protocols were approved by IACUC committees of UC-Santa Cruz (protocol #20061056–122204) the George Washington University (protocol #A186), and Rutgers University (11–030).

### Ecological Data Collection

#### Fruit availability

Monthly phenology data collection at each site started in 2003 using near identical methods [71,73,76]. All trees with a diameter at breast height (DBH) of >10cm were monitored each month for the presence/abundance of fruit. An average of 1344 trees covering 2.4 ha in Sabangau and 1868 trees covering 2.3 ha in Tuanan were monitored. Tree species were identified by skilled local botanists and consistency of identifications across sites was checked by cross visits of researchers from both sites [71]. A fruit availability index (FAI) was calculated as the percentage of fruiting trees in the plots each month, and used as a proxy for forest productivity. To examine site differences in the availability of fruit and energy intake among the high and low fruit periods, monthly FAI scores were converted to z-scores and months with z < -1 were assigned into the low category, -1 ≤ z ≤1were assigned to the medium category, and z > 1were assigned to the high fruit category [following 3]. Stem density (number of stems per hectare) of matched species was also calculated from phenology plots at each site [71].

#### Plant sample collection, nutritional analyses and energy intake calculations

Food samples were collected from July 2005-June 2007 in Sabangau and from June 2004-September 2010 in Tuanan by climbing trees and/or by collecting fresh fruit that had fallen from the tree. Wherever possible, samples were collected from trees in which orangutans had been observed feeding, were matched for the stage of ripeness that the orangutans consume, and were collected from multiple trees (2–10) to account for variation in nutrient content between trees of the same species [77]. Following previous terminology [52], we refer to a specific food species-part combination as a “food item”.

Samples were brought back to camp that day and processed [28,29,52]. We counted the number of pieces of each food item in the collected samples for each species (area for inner-bark), separated samples into parts ingested and discarded based on our observations of orangutan feeding at each site, and then dried the ingested parts of each sample at 30–50°C. Once dry, samples were weighed to obtain a field-dry weight. The field dry-weight per item was calculated then by dividing this value by the number of items in the original sample. Dried samples were stored with silica gel until they were shipped for nutritional analyses.

All nutritional analyses on plant foods from each site were conducted in the Laboratory of Nutrition Testing, Research Center for Biology, Indonesian Institute of Sciences (LIPI), Cibinong—Bogor, Indonesia following [52], unless noted below. Crude protein was determined using the Kjeldahl procedure for total nitrogen, which was then multiplied by 6.25 [78]. Crude lipid content was determined by the Soxtec method using Soxtex System HT2 as described in [79]. The detergent system of fiber analysis was used to quantify neutral-detergent fiber (NDF) [80,as modified by 81] and total non-structural carbohydrates (TNC) were calculated by difference [28]. We used published values for invertebrate (termite and ant) nutritional composition [82,83]. Dry matter (DM) and organic matter (OM) were calculated following [28]. All results are reported as the percentage of organic matter (OM).

Standard conversion factors were applied to the nutritional fractions to calculate metabolizable energy content per food item (ME kcal/100g OM = (4 x %TNC) + (4 x %CP) + (9 x %lipids) + (0.543 x %NDF), using the lower NDF digestion coefficient as recommended by [28]. Studies focusing on orangutan energetics have used different digestion coefficients for NDF [28,29,52]. In a digestion and passage experiment on chimpanzees, Milton and Demment [84] found that chimpanzees digested 54.3% of the NDF fed to them from biscuits containing 34% NDF, indicating a digestion coefficient of 0.543. This, value of 34% is the same as the average NDF reported for orangutan foods consumed in the wild [28]. Although we opted for use of the lower digestion coefficient in this study, it is important to note that analyses run with the higher digestion coefficient [cf. 52], did not alter the direction or significance of results (see S2 Table). Metabolizable energy per plant item was then calculated by multiplying the amount of energy per 100g OM by the dry weight of the item.

Energy intake per feeding bout was calculated following [52]. Feeding bouts were summed across the day to obtain total daily caloric intake (Kcal). If nutritional data and/or feeding rates were not available for a given plant species, we took either 1) data from a species of the same genus that was similar in fruit size/character, or where this was not available 2) the average value for a given item category (fruit, leaves, inner-bark, flowers, vegetation) and stage of maturity. This accounted for only 19.4% of the feeding bouts (3,542 out of 18,235) and 14.7% (100,842 out of 684,348) of feeding minutes for Tuanan and 29.6% (2,951 out of 9,885) feeding bouts and 19.1% (38,218 out of 199,675) of feeding minutes for Sabangau. Nutritional analyses were conducted on 221 species/parts from Tuanan and 183 from Sabangau [85].

### Data analyses

To examine site differences in nutritional and energetic composition (ME) of all dietary items for which nutritional data were available set at each site, Wilcoxon Signed Rank Tests were used. The species included in our full dataset include the most commonly consumed items over the study period and over 80% of items in the diet at each site. To examine variation in nutritional composition, intake rates (energy and item), and availability among the same species/item combinations consumed at the two sites, matched Wilcoxon rank tests with continuity correction were used. Orangutans may consume several different items from the same species and thus for the matched species analyses we matched by food items. Because our goal was to compare the same individual food items found at each site, we did not use models with individual ID as a random effect as matched tests currently do not incorporate random effects. Thus, to reduce the potential effect of individual ID, we used feeding rates and energy intakes for adult females only for these matched items. Variation in fruit availability at the two sites was examined using T-tests matched by year and month.

To examine overall site differences in daily caloric intake, total feeding time, and feeding time on fruit, we used Generalized Additive Mixed Models (GAMM) in the lme4 packages of R v3.1.1 [86,87]. Fixed effects in the model included field site, sex/age class (independent nulliparous female, adult female (primiparous or multiparous), adult flanged male, adult unflanged male), and the percentage of fruiting trees. Orangutan identification was included as a random effect in each model. Total follow time or total feeding time was included as an offset function in the GAMM. In addition to the overall GAMMs, we further explored the data using GLMMs by breaking fruit availability into categories (high, middle, low; see above) to test if our results held during only the low fruit periods when we would expect the animals are most energetically stressed. All test results are reported as two-tailed and significance was set at α ≤ 0.05.

## Results

**Fig 1:**
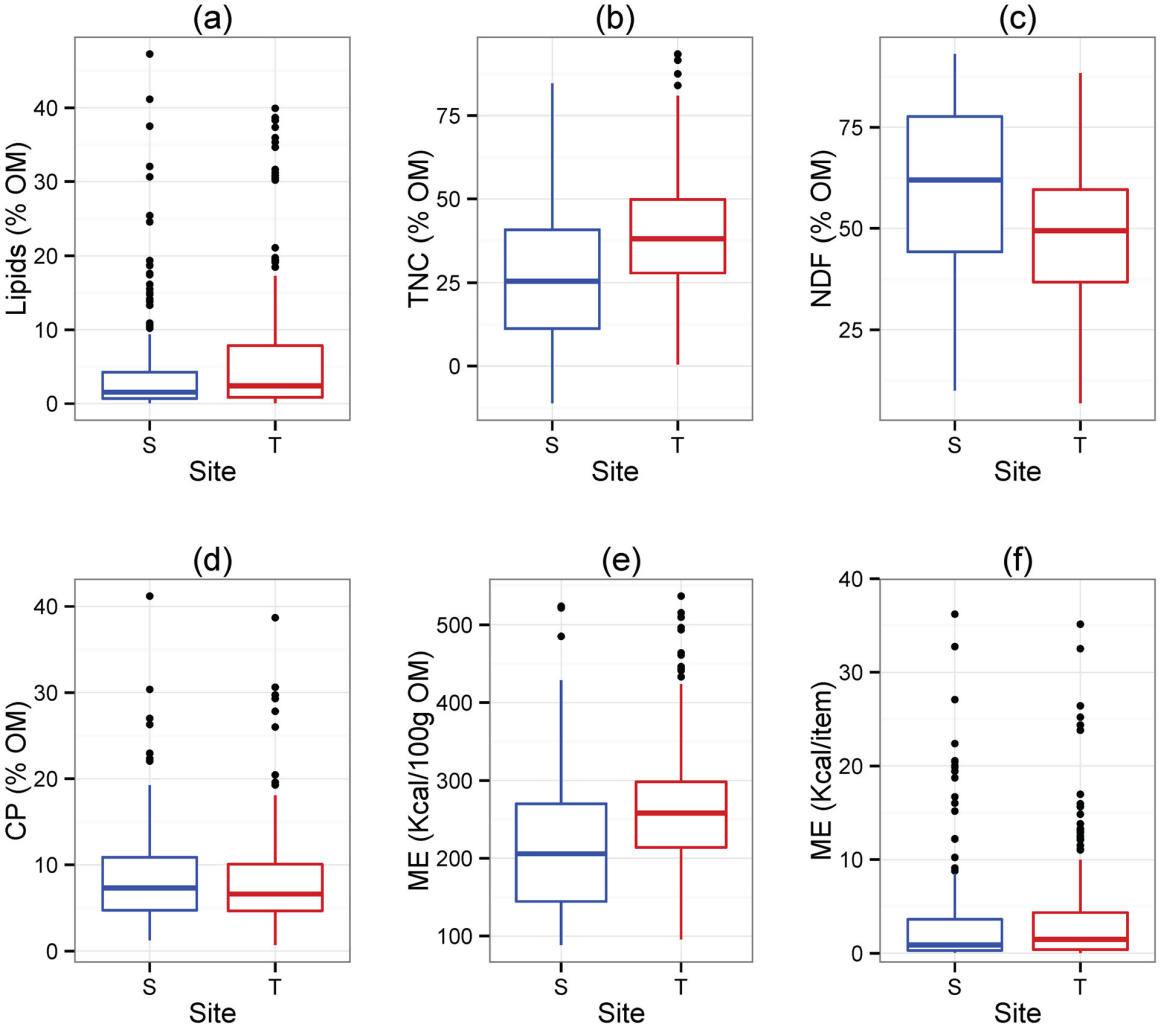
Variation in macronutrient composition in the orangutan diets in Tuanan (T) and Sabangau (S). All comparisons are based on organic matter (OM) and Wilcoxon Signed Rank Test were used. A significant negative Z-statistic indicates Tuanan is greater. (a) Lipids (Z = -2.17, p = 0.03); (b) Total non-structural carbohydrates (TNC; Z = -5.43, p < 0.0001); (c) Neutral detergent fiber (NDF; Z = 5.63, p < 0.0001); (d) Crude protein (CP; Z = 0.64, p = 0.52); (e) Metabolizable energy (Kcal/100 g OM) (Z = -5.55, p < 0.0001); (f) Metabolizable energy/item (Kcal)(Z = -2.64, p = 0.008). See Table 1 for complete statistical results.

The overall sampled diet in Tuanan was higher in lipids and total non-structural carbohydrates (TNC), while the diet in Sabangau was higher in neutral detergent fiber (NDF) (Fig 1a–1c; Table 1); there was no difference in crude protein between plants consumed at the two sites (Fig 1d, Table 1). This resulted in higher metabolizable energy (Kcal per 100g OM) and metabolizable energy per plant item (Kcal) at Tuanan (Fig 1e and 1f; Table 1).

**Table 1:** Variation in entire sampled diet in Sabangau (S) and Tuanan (T). Wilcoxon Signed Rank Test were used to compare macronutrients and metabolizable energy (ME) (expressed as % OM) between the two sites. TNC = Total non-structural carbohydrates; CP = Crude Protein; NDF = Neutral Detergent Fiber; ME = metabolizable energy Means and standard errors along with sample size (n) are reported.

	Tuanan	Sabangau	Stat	p-value
	mean ± SE (n)	mean ± SE (n)		
Lipids	5.59 ± 0.54 (219)	4.38 ± 0.59 (183)	Z = -2.17	p = 0.03
TNC	38.42 ± 1.28 (222)	28.15 ± 1.4 (183)	Z = -5.43	p < 0.0001
CP	8.18 ± 0.38 (221)	8.43 ± 0.42 (183)	Z = 0.64	p = 0.52
NDF	48.29 ± 1.28 (219)	59.20 ± 1.4 (183)	Z = 5.63	p < 0.0001
ME (Kcal/100g OM)	263.01 ± 5.74 (219)	217.85 ± 6.28 (183)	Z = -5.55	p < 0.0001
ME (per plant item)	2.95 ± 0.40 (217)	2.53 ± 0.45 (177)	Z = -2.64	p = 0.0082

**Fig 2:**
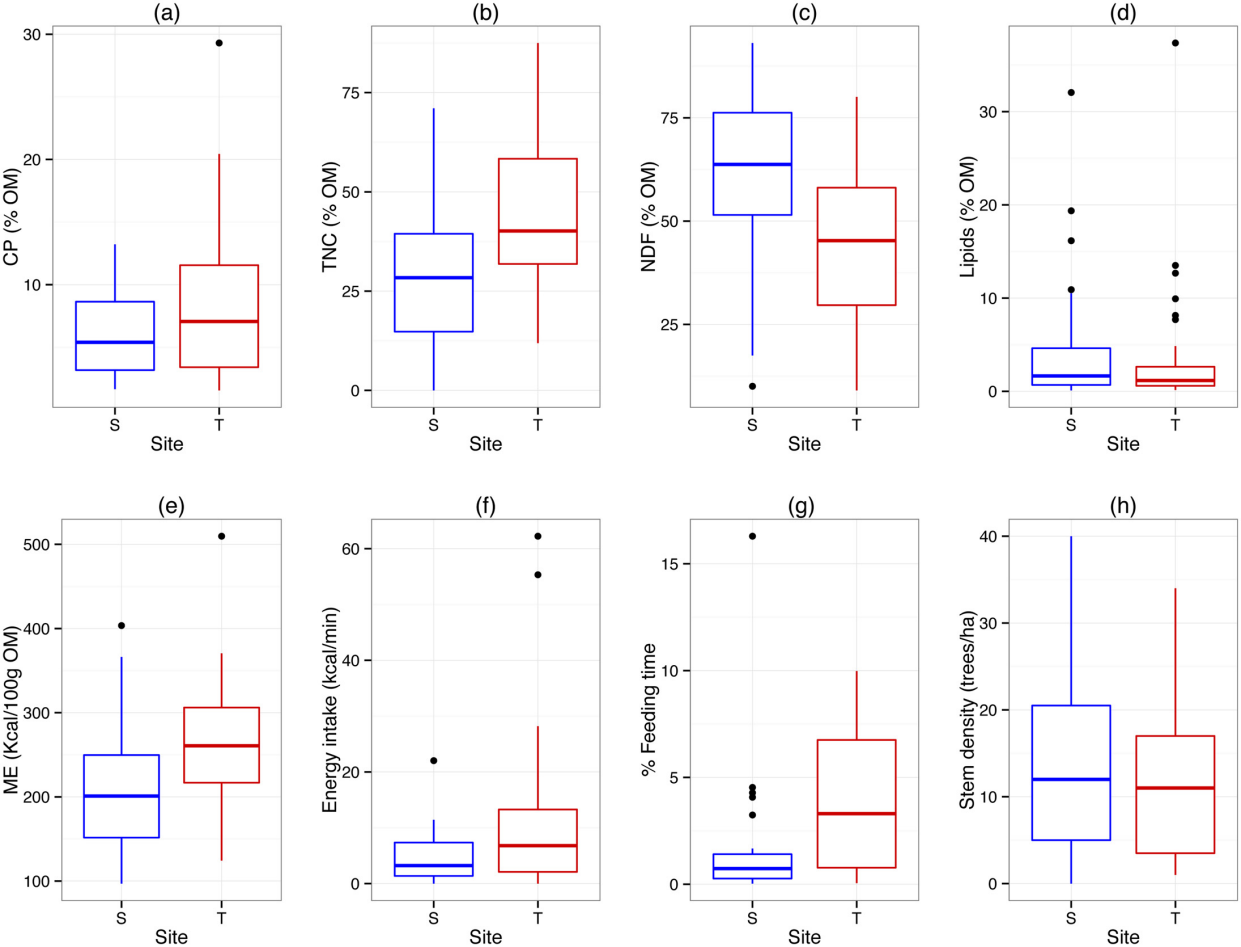
Matched plant species comparisons of dietary items in Tuanan (T) and Sabangau (S). All macronutrient and energy comparisons are based on organic matter (OM) and Matched Wilcoxon Rank Tests were used. A negative S-statistics indicates Tuanan is greater. (a) Crude protein (S = -207, p = 0.003); (b) TNC (S = -284, p < 0.0001); (c) NDF (S = 291, p < 0.0001); (d) Lipids (S = 109, p = 0.13); (e) Metabolizable energy (Kcal/100g OM)(S = -235, p = 0.0005; (f) Energy intake (Kcal/min) (S = 52.5, p = 0.049); (g) Percentage of time feeding on plant items (S = -106, p = 0.001). See Table 2 for complete statistical results.

Comparison of the nutritional composition of 39 food items from 22 species that were consumed at both sites and for which matching data were available revealed that matched items in Tuanan were higher in crude protein and TNC, and lower in NDF; there was no difference in lipids among sites (Fig 2a–2d, Table 2). Metabolizable energy (Kcal per 100g OM) of matched items was higher at Tuanan compared to Sabangau, resulting in higher energy intake (Kcal/min) on these items (Fig 2e and 2f; Table 2). In other words, orangutans eating the same amount of a given food item receive higher energy intake at Tuanan compared to Sabangau. This higher energy intake was not due to differences in feeding rates or field dry weight (g) of these plant items (Table 2), and thus reflects the energy available in these matched items. The orangutans in Tuanan spent a greater percentage of their daily feeding time on these matched items compared to Sabangau orangutans (Fig 2g; Table 2), and this was not due to difference in stem density of these species (Fig 2h; Table 2).

**Table 2:** Variation in macronutrients, metabolizable energy (ME) (expressed as % OM), and measures of availability in matched plant items in Sabangau (S) and Tuanan (T). Matched Wilcoxon Rank Tests were used to compare the two sites. TNC = Total non-structural carbohydrates; CP = Crude Protein; NDF = Neutral Detergent Fiber; ME = metabolizable energy. Means and standard errors along with sample size (n) are reported (n = 39 unless noted).

	Tuanan	Sabangua	Stat	p-value
	mean ± SE	mean ± SE		
Lipids	3.38 ± 1.04	4.33 ± 1.02	S = 109, df = 38	p = 0.13
TNC	43.25 ± 2.91	28.9 ± 3.09	S = -284, df = 38	p < 0.0001
CP	8.266 ± 1.01	6.185 ± 0.55	S = -207, df = 38	p = 0.003
NDF	45.09 ± 2.92	60.9 ± 3.16	S = 291, df = 38	p < 0.0001
ME (Kcal/100g OM)	261.04 ± 12.2	212.34 ± 12.31	S = -235, df = 38	p = 0.0005
Energy intake/min	10.87 ± 2.32	5.07 ± 1.09	S = -52.5, df = 20	p = 0.049
Feeding rate (item/min)	13.13 ± 2.69	9.77 ± 2.04	S = -30.0, df = 18	p = 0.24
Field dry mass (g)	1.56 ± 0.73	1.57 ± 0.55	S = 45, df = 38	p = 0.54
% of feeding time	4.06 ± 0.64	1.78 ± 0.62	S = -106, df = 23	p = 0.001
Stem density (trees/ha)	12.1 ± 2.22	15.11 ± 3.15	S = 26, df = 18	p = 0.26

**Fig 3:**
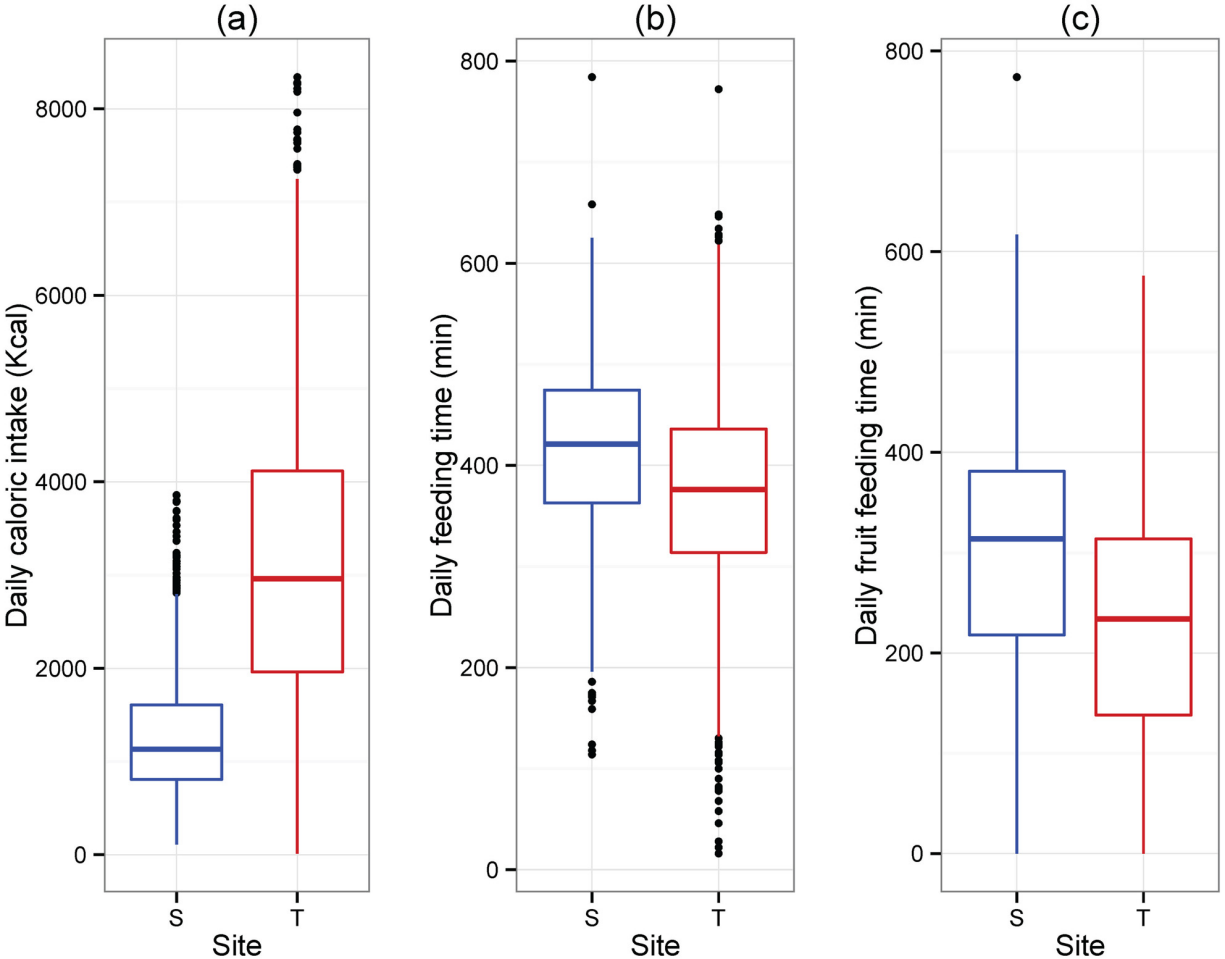
Daily caloric intake (a), total feeding time (b), and fruit feeding time compared (c). Site differences in (a) Daily caloric intake (t-statistic = 12.26, p < 0.0001), (b) Total feeding time (t-statistic = -6.35, p < 0.0001), and (c) Fruit feeding time (t-statistic = -7.19, p < 0.0001) between the two sites. T = Tuanan, S = Sabangau. Generalized additive mixed models (GAMM) were used. A positive t-statistic indicates Tuanan is higher. See Table 3 for models and full statistical results.

While these data clearly indicate that the nutritional quality of dietary items is greater in Tuanan, how this translates into daily energy intake is likely to have direct impacts on fitness. Daily caloric intake was significantly greater in Tuanan compared to Sabangau (Fig 3a; Table 3a). This was not due to the Tuanan orangutans spending more time feeding per day (Fig 3b; Table 3b); indeed, they actually spent less time feeding on fruit (Fig 3c, Table 3c). This pattern held during the low-fruit periods: Tuanan orangutans had greater caloric intake, but spent less time feeding on fruit compared to the Sabangau orangutans (Table 4). During the high-fruit periods, Tuanan orangutans consumed more energy and spent less time feeding, but they did not spend less time feeding on fruit as they did during the low fruit periods (Table 4).

**Table 3:** Site variation in (a) Daily caloric intake, (b) Daily feeding time (min), and (c) Daily time spent feeding on fruit (min). All models include the variable age/sex class: FLM = flanged male; NUF = nulliparous independent female; UFM = unflanged adult male; AF = adult female; Age/Sex class comparisons are against AF. FAI = Percentage of fruiting trees.

(a) GAMM of Daily Caloric Intake (Kcal of OM); Random Effect = Orangutan ID;						
Model offset = length of follow (min); r^2^_adj_ = 0.25; n = 2826						
Variable	mean ± SE	n	estimate	SE	t-stat	p-value
**Site**			1689.66	137.81	12.26	<0.0001
Tuanan	3124.0 ± 33.2	2208				
Sabangau	1304.6 ± 28.9	624				
**FAI**			119.95	9.71	12.35	<0.0001
**Age/Sex Class**						
FLM			-237.007	156.29	-1.516	0.13
NUF			-590.17	137.89	-4.28	<0.0001
UFM			-55.8	175.22	-0.318	0.75
(b) GAMM of Daily Feeding Time (min); Random Effect = Orangutan ID;						
Model offset = length of follow (min); r^2^_adj_ = 0.25; n = 2868						
Variable	mean ± SE	n	estimate	SE	t-stat	p-value
**Site**			-82.17	12.94	-6.35	<0.0001
Tuanan	373.47 ± 1.96	2233				
Sabangau	414.93 ± 3.48	635				
**FAI**			-4.055	0.61	-6.6	<0.0001
**Age/Sex Class**						
FLM			10.31	15.04	0.69	0.49
NUF			9.7	9.89	0.98	0.33
UFM			16.32	16.7	0.978	0.33
(c) GAMM of Daily Feeding Time on Fruit (min); Random Effect = Orangutan ID;						
Model offset = daily feeding time (min); r^2^_adj_ = 0.18; n = 2862						
Variable	mean ± SE	n	estimate	SE	t-stat	p-value
**Site**			-89.98	12.52	-7.19	<0.0001
Tuanan	227.61 ± 2.52	2233				
Sabangau	294.29 ± 5.02	635				
**FAI**			12.65	0.81	15.57	<0.0001
**Age/Sex Class**						
FLM			-9.33	14.29	-0.653	0.51
NUF			-21.05	11.9	-1.77	0.08
UFM			7.6	15.98	0.48	0.63

**Table 4:** Variation in daily energy intake (Kcal), daily feeding time (minute), and daily fruit feeding time (minutes) during low, medium, and high fruit periods. GMM were used to compare the two sites and included orangutan ID as a random effect. Data represent mean ± SE.

	Tuanan	Sabangau	model	Tuanan	Sabangau	model	Tuanan	Sabangau	model
	Low	Low		Medium	Medium		High	High	
	(n = 362)	(n = 82)		(n = 1626)	(n = 433)		(n = 245)	(n = 121)	
Daily caloric intake (kcal)	1989.67 ± 55.39	1170.92 ± 54.54	r^2^_adj_ = 0.22	3279.23 ± 39.26	1293.87 ± 35.3	r^2^_adj_ = 0.8	3776.47 ± 81.08	1431.51 ± 75.35	r^2^_adj_ = 0.56
			t-stat = -3.76			t-stat = -13.10			t-stat = -10.85
			p = 0.0006			p<0.0001			p<0.0001
Daily feeding time (min)	366.33 ± 4.53	435.14 ± 8.31	r^2^_adj_ = 0.39	376.22 ± 2.34	418.88 ± 4.27	r^2^_adj_ = 0.20	356.63 ± 5.43	387.27 ± 7.85	r^2^_adj_ = 0.26
			t-stat = 3.89			t-stat = 6.08			t-stat = 2.46,
			p = 0.0005			p<0.0001			p = 0.02
Daily feeding time on fruit (min)	109.31 ± 4.63	216.57 ± 14.34	r^2^_adj_ = 0.34	246.68 ± 2.82	310.82 ± 5.81	r^2^_adj_ = 0.10	275.84 ± 5.27	287.14 ± 11.47	r^2^_adj_ = 0.29
			t-stat = 4.59			t-stat = 7.13			t-stat = 1.07
			p<0.0001			p<0.0001			p = 0.29

**Fig 4:**
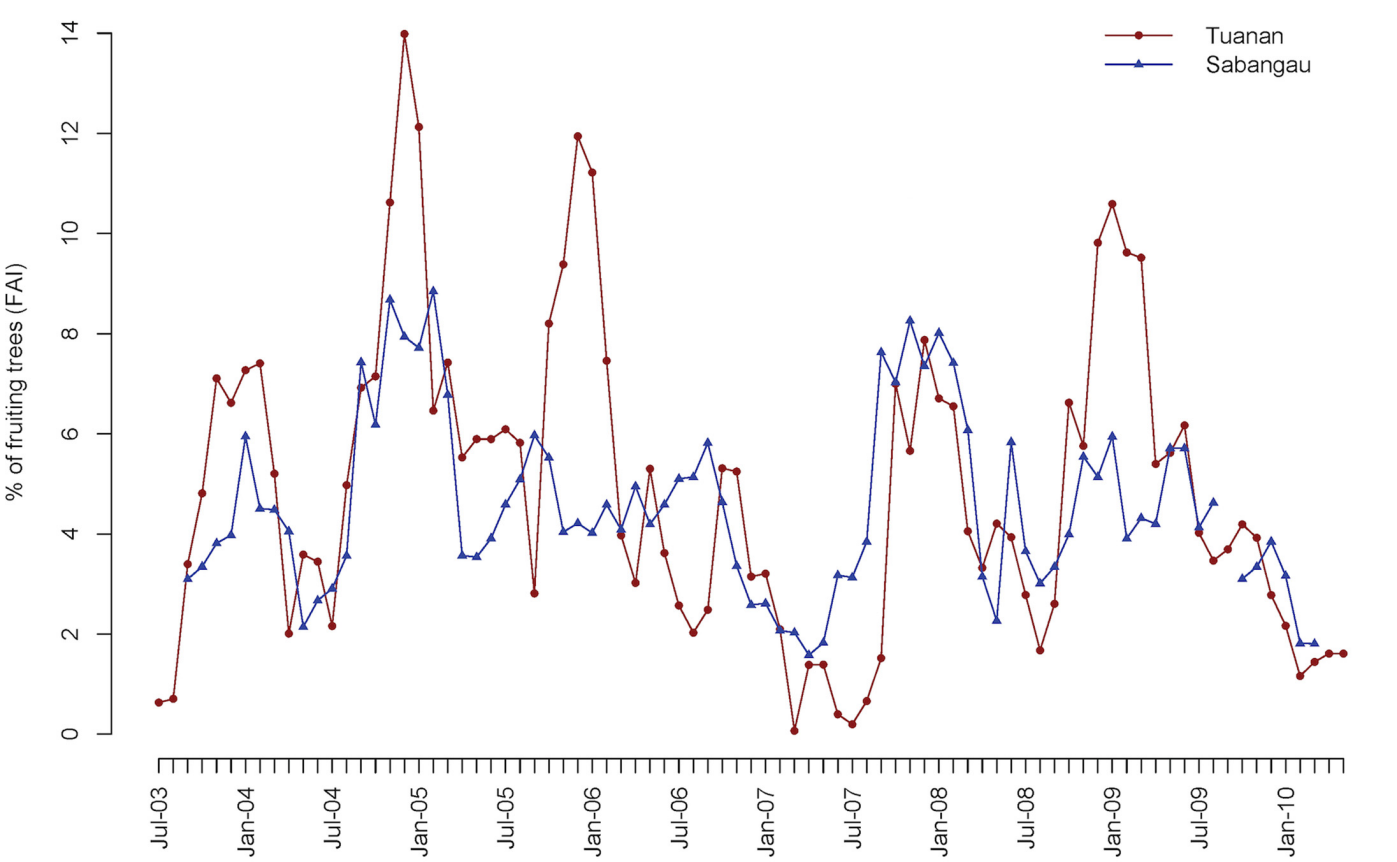
Percentage of fruiting trees over the entire study period in Tuanan (red) and Sabangau (blue).

Longer fruit feeding time in Sabangau was not a consequence of higher productivity, as fruit availability in Tuanan was marginally higher relative to Sabangau (Matched Paired t-test, t = 1.93, p = 0.05, df = 77; Fig 4; mean Tuanan = 4.92 ± 3.08, mean Sabangau = 4.54 ± 1.77). Although fruiting was correlated between the two sites (Spearman ρ = 0.58, p<0.0001), fluctuations in fruit availability were more extreme in Tuanan in both directions, with higher peaks (Wilcoxon Matched Sign Rank Test, Z = 3.84, p<0.0001, n = 11,12) and lower troughs of fruit availability in Tuanan (Wilcoxon Matched Sign Rank Test, Z = -3.84, p<0.0001, n = 10,11). However, the duration, in number of months, of the high and low fruit periods did not differ between the sites (Wilcoxon rank sums (high: Χ^2^ = 0.26, df = 1, p = 0.61; low Χ^2^ = 0.0045, df = 1, p = 0.95).

## Discussion

Overall, our predictions were supported: the plant items consumed by the orangutans in Tuanan were higher in nutritional quality, resulting in higher daily caloric intake. This result was consistent for both the overall diets consumed at both sites and for those items for which we had matched nutritional data. Orangutans in Sabangau spent a greater amount of time feeding on fruit, which has a higher energetic return compared to other items in their diet [28,29,52], but they still had lower daily energy intake compared to orangutans in Tuanan. Thus, it is likely that the Sabangau orangutans spend more time feeding on fruit, a resource with higher caloric gains per unit feeding time [85,88,89]), in an attempt to ‘make up for’ their typically lower quality diet [52]. In agreement with this, Harrison et al. [52] found no correlation between fruit availability, energy intake, and ketone production in the Sabangau orangutan population (with the exception of a fairly weak positive relationship between fruit availability and energy intake in flanged males, *r* ^*2*^ = 0.162) and showed that these orangutans experienced prolonged periods of energy shortfalls. Conversely, in Tuanan, orangutans fall into a negative energy balance state during periods of low fruit availability when energy intake is lowest, as evidenced by significantly greater levels of ketone production, but not during high fruit periods [53]. During the low fruit period, Tuanan orangutans, on average, consumed 819 more calories per day compared to orangutan in Sabangau (Table 4). This difference was even more extreme during the high fruit periods, with Tuanan orangutans consuming 2345 more calories per day during the (Table 4). This may have severe consequences for the Sabangau orangutans: total daily energy expenditure exceeds energy intake during on average 84% of the months during the four years between 2003–2007 [52], whereas for Tuanan total daily energy expenditure exceeds energy intake for about 20% of the months [53,90]. Thus, during the high fruit periods, the Tuanan orangutans appear to be better able to build fat reserves that they can catabolize during the low fruit periods when they fall into a negative energy balance state. This places energy intake levels in Tuanan between Sabangau and Gunung Palung in West Kalimantan [29,52,88].

Differences in forest productivity in terms of overall fruit availability were not as apparent, although the peak fruit periods were higher in Tuanan, reaching up to 14% of stems sampled within a month compared to 9% at Sabangau. However, when fruit availability was low, it was also at lower levels in Tuanan. Thus, fruiting is more extreme in both directions in Tuanan. These differences are unlikely to be driven by climatic variation between the two sites, as rainfall and temperature comparisons revealed high similarities [68]. Harrison et al. [68] found significant variation in the onset of fruiting and flowering events both within and among species at the two sites, despite their close geographic proximity. Finally, other studies focusing on the relationship between nutrition and fruit availability have used the energy of fruits available/hectare as a measure of food availability [17,48,91]. We used FAI instead of this measure because this measure is more reliably consistent across researchers collecting phenology data at both sites and Harrison [85] found that FAI had a stronger effect on orangutan energy intake than did fruit-energy availability in Sabangau.

While both Tuanan and Sabangau are classified as peat-swamp forests, the ecological differences between the two sites, namely the shallower peat and a more alluvial flooding regime in Tuanan, likely contribute to the differences in plant nutrient availability and, hence, nutritional composition of orangutan foods, fruiting patterns, and overall fruit availability [see also 68]. Future studies should examine variation in mineral composition of the peat soils at the two sites and how these may be linked to this observed variation in plant nutritional quality.

The availability of fruit resources is considered a major factor in determining vertebrate frugivore densities and carrying capacities [11,15,16,92]. Recent studies have emphasized the value of examining the relationship between food availability and vertebrate population density on a small spatial scale, as ecological conditions are less likely to differ between neighboring populations in climatically similar areas, and thus any relationships that may exist are more likely to become apparent [11,93]. It is likely that the higher orangutan population density in Tuanan [56,69] is linked to higher nutritional quality of plant species and the resulting higher daily orangutan caloric intake observed in this study.

However, additional factors may also influence population density at the two sites. Both logging and particularly hunting have been demonstrated to affect orangutan population densities [12,14,55,56,94,95]. Tuanan and Sabangau both experienced legal and illegal logging disturbances prior to 2004 [58,69]. Although quantitative data on the intensity of these threats are not available, because Sabangau is closer to Central Kalimantan’s capital, Palangka Raya (11 km versus 58 km), it may have experienced higher intensities of logging and hunting. It is therefore probable that these historical processes, combined with lower food nutrient content and overall fruit availability, explain the lower orangutan population density in Sabangau.

A second line of evidence also supports a higher quality habitat in Tuanan relative to Sabangau: differences in orangutan home range characteristics. While overall adult female home range size does not appear to differ between Tuanan and Sabangau [96–99], both overall home range and home range core areas (50% use) of females overlap more in Tuanan compared to Sabangau [97,99,100]. Specifically, related adult female home range overlap in Tuanan is 57% and core area overlap is 15%, compared to 38% and 0% overlap in Sabangau [97,100]. This suggests that, while adult females live at higher densities in Tuanan, they may experience less competition for nutrients, and thus can afford to have more overlapping home ranges. This finding concurs with a larger scale study that also found a positive relationship between primate density and home range overlap, which is expected in the absence of active territory defense [101].

The relatively solitary lifestyle, late weaning, and overall slow life history of orangutans is likely a consequence of the ecological conditions characteristic of Southeast Asian tropical forests [29,43,44,102,103] [; but see [104]]. These forests have overall low mean productivity and great inter-annual variation in productivity [3,57,105,106]. Currently, the data on comparative reproductive ecology of wild orangutans are few and sufficient data sets with complete inter-birth intervals from Sumatra and Borneo remain limited [see [47] for a review]. While we currently do not have a sufficient comparative sample with complete IBIs and reproductive rates from the two field sites, it will be interesting to test if reproductive rates and ultimately fitness are higher in Tuanan compared to Sabangau as our sample size increases with future observations.

## Conclusions

In summary, our data support a link between bottom-up regulatory factors, variation in nutritional intake of a mostly frugivorous mammal species, and population density. This study has implications for the conservation of viable habitat for endangered frugivores like orangutans. Peat-swamp forests hold the largest remaining relatively undisturbed populations of Bornean orangutans (*Pongo pygmaeus*) and are considered excellent habitats for orangutans [56,58,60]. As more orangutans are relocated due to agricultural pressures, specifically the growing pressures of oil palm expansion in peatland habitats [107,108], finding suitable habitats that provide sufficient energy for orangutans has become an increasing challenge [109]. Here, we have shown that what appears to be similar habitat does not necessarily translate into similar nutrient intake of its inhabitants. Our results indicate that reductions in habitat quality that would be expected following human disturbance, and consequently the availability of higher-quality fruits, are likely to lead to negative impacts on orangutan nutrition, with potential long-term impacts on population density. This suggests that it is important to incorporate careful habitat quality assessment and maintenance into future conservation action plans for this endangered species.

## Supporting Information

S1 Fig Map of Tuanan Orangutan Research Project area and the Sabangau Study area, Natural Laboratory of Peat-swamp Forest (NLPSF). Reprinted from [68] under a CC BY license, with permission from the Assoc. for Tropical Biology and Conservation original copyright 2015.
(PDF)

S1 Table Breakdown of the number of individuals in each sex class in this study. (PDF)

S2 Table Site variation in daily caloric intake (using the high NDF coefficient of 1.6); FLM = flanged male; NUF = nulliparous independent female; UFM = unflanged adult male; AF = adult female; Age/Sex class comparisons are against AF. FAI = Percentage of fruiting trees.
(PDF)
