# Supplementary material for: Nutritional Differences between Two Orangutan Habitats: Implications for Population Density
Source: PLoS One. 2015 Oct 14;10(10):e0138612. doi: 10.1371/journal.pone.0138612 (PMC4605688; doi:10.1371/journal.pone.0138612)

S1 Figure. Map of Tuanan Orangutan Research Project area and the Sabangau Study area, Natural Laboratory of Peat-swamp Forest (NLPSF). Reprinted from Harrison et al. (2015) under a CC BY license, with permission from the Assoc. for Tropical Biology and Conservation, original copyright 2015.

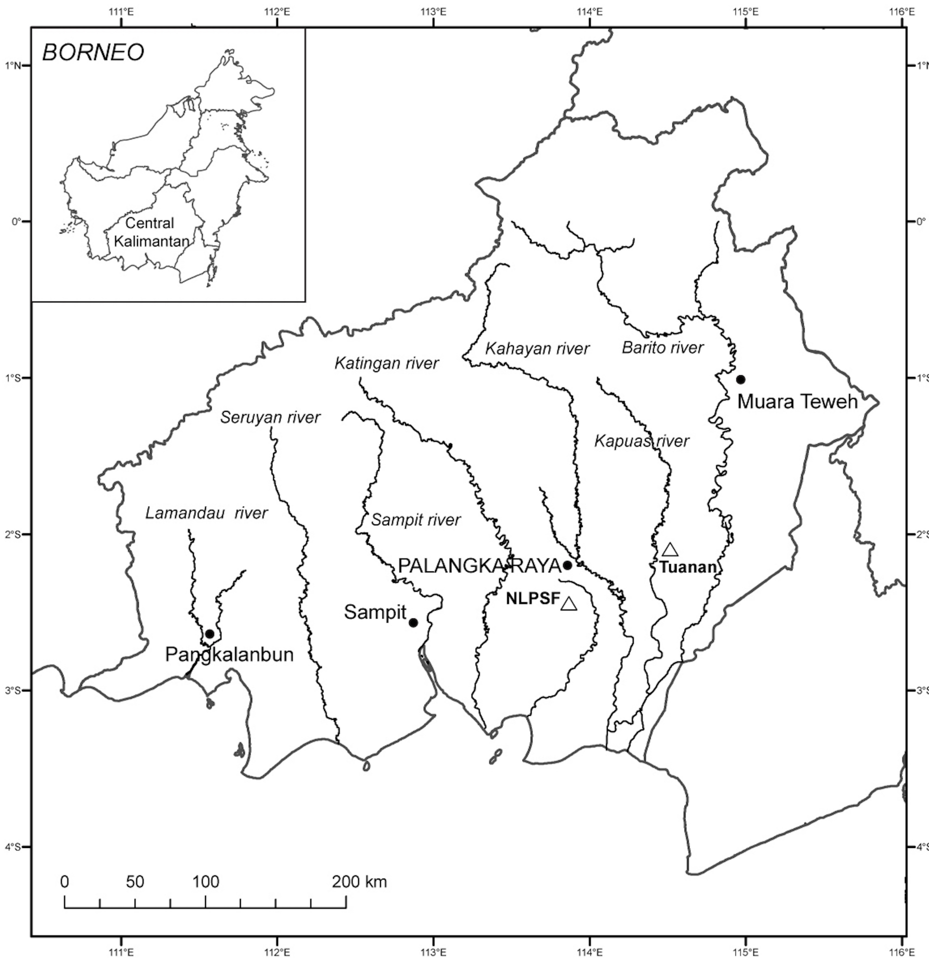

Supplement: S1 Fig — Reprinted from [68] under a CC BY license, with permission from the Assoc. for Tropical Biology and Conservation original copyright 2015. (PDF) [file pone.0138612.s001.pdf]
