# Supplementary material for: Nutritional Differences between Two Orangutan Habitats: Implications for Population Density
Source: PLoS One. 2015 Oct 14;10(10):e0138612. doi: 10.1371/journal.pone.0138612 (PMC4605688; doi:10.1371/journal.pone.0138612)
Supplement: S1 Table — (PDF) [file pone.0138612.s002.pdf]

S1 Table. Breakdown of the number of individuals in each sex class in this study

	Sabangau	Tuanan
Total Follow Hours 2003-2010	6976.9	25504.7
Adult females	6	12
Nulliparous independent females	5	6
Flanged males	13	21
Unflanged males	9	12
Number of births recorded since studies started (June 2003)	8 from 5 females	16 from 12 females
