# Supplementary material for: Nutritional Differences between Two Orangutan Habitats: Implications for Population Density
Source: PLoS One. 2015 Oct 14;10(10):e0138612. doi: 10.1371/journal.pone.0138612 (PMC4605688; doi:10.1371/journal.pone.0138612)
Supplement: S2 Table — FAI = Percentage of fruiting trees. (PDF) [file pone.0138612.s003.pdf]

S2 Table: Site variation in daily caloric intake (using the high NDF coefficient of 1.6); FLM= flanged male; NUF = nulliparous independent female; UFM = unflanged adult male; AF = adult female; Age/Sex class comparisons are against AF. FAI = Percentage of fruiting trees

GAMM of Daily Caloric Intake (Kcal of OM); Random Effect = Orangutan ID;
Model offset = length of follow (min); r^2 adj = 0.23; n=2826

Variable	mean ± SE	n	estimate	SE	t-stat	p-value
**Site**			1903.49	165.55	11.49	<0.0001
Tuanan	3748.36	2208				
Sabangau	1707	624				
**FAI**			150.46	11.68	12.89	<0.0001
**Age/Sex Class**						
FLM			-223.83	187.74	-1.192	0.233
NUF			-669.04	165.4	-4.045	<0.0001
UFM			-66.84	210.58	-0.317	0.751
